# Direct electric field control of the skyrmion phase in a magnetoelectric insulator

**DOI:** 10.1038/s41598-018-27882-4

**Published:** 2018-07-11

**Authors:** A. J. Kruchkov, J. S. White, M. Bartkowiak, I. Živković, A. Magrez, H. M. Rønnow

**Affiliations:** 1 Department of Physics, Harvard University, Cambridge, MA 02138 USA; 2 Laboratory for Quantum Magnetism (LQM), Insititute of Physics, École Polytechnique Fédérale de Lausanne (EPFL), CH-1015 Lausanne, Switzerland; 3 Laboratory for Neutron Scattering and Imaging (LNS), Paul Scherrer Institut (PSI), CH-5232 Villigen, Switzerland; 4 Laboratory for Scientific Developments and Novel Materials (LDM), Paul Scherrer Institut (PSI), CH-5232 Villigen, Switzerland; 5 Crystal Growth Facility, Insititute of Physics, École Polytechnique Fédérale de Lausanne (EPFL), CH-1015 Lausanne, Switzerland

## Abstract

Magnetic skyrmions are topologically protected spin-whirls currently considered as promising for use in ultra-dense memory devices. Towards achieving this goal, exploration of the skyrmion phase response and under external stimuli is urgently required. Here we show experimentally, and explain theoretically, that in the magnetoelectric insulator Cu_2_OSeO_3_ the skyrmion phase can expand and shrink significantly depending on the polarity of a moderate applied electric field (few V/*μ*m). The theory we develop incorporates fluctuations around the mean-field that clarifies precisely how the electric field provides direct control over the free energy difference between the skyrmion and the surrounding conical phase. The quantitative agreement between theory and experiment provides a solid foundation for the development of skyrmionic applications based on magnetoelectric coupling.

## Introduction

To realise skyrmion-based applications (skyrmionics)^1–5^, research into the creation, control and stabilisation of skyrmions is in an active phase^1,6–13^. In this context, it could seem problematic that in bulk materials the skyrmion phase is stable only for a narrow interval at finite temperature (*T*) just below the magnetic ordering temperature *T*_*C*_, and under an applied magnetic field (*H*)^6,14–17^. In Cu_2_OSeO_3_ for example, the skyrmion phase spreads down in *T* by just 3.5% of *T*_*C*_, occupying no more than 1% of the total ordered phase space that is otherwise dominated by topologically trivial helical or conical phases^7,17,18^. This limited skyrmion phase space is observed also in other known bulk hosts of skyrmions^14–16,19^. On the other hand, the finite extent of the skyrmion phase pocket can be considered to present an interesting advantage, since relatively small perturbations can dramatically influence the skyrmion phase stability. It follows that the ability to enhance or suppress the skyrmion phase space in a sample can provide a flexible platform for the respective creation or destruction of skyrmions, a process that can be technologically useful. Here we describe a simple and reliable mechanism for the stabilisation and destabilisation of the skyrmion lattice (SkL) phase by exploiting electric (*E*) fields applied to the insulating magnetoelectric material Cu_2_OSeO_3_.

To date, several approaches for skyrmion manipulation have been demonstrated that make use of either moderate electric currents, electric fields, or thermal gradients^6,8–13,20–23^. In addition, progress towards tuning the bulk skyrmion phase stability was also demonstrated using both applied uniaxial^24,25^ and hydrostatic pressure^18^. For possible applications however, the use of *E* fields to manipulate skyrmions and skyrmion phase stability in insulators offers several potential advantages for applications, yet this approach remains still little-explored experimentally^6,8,23,26^. Moreover, the *E* field control of the SkL phase stability remains an outstanding theoretical issue.

Here we report a combined experimental and theoretical study of skyrmion phase stability under moderate *E*-fields (V/*μ*m) in the model insulating skyrmion host Cu_2_OSeO_3_. We use the microscopic probe of small-angle neutron scattering (SANS) to show that in Cu_2_OSeO_3_, the extent of the skyrmion phase stability either expands or shrinks in both *T* and *H*, dependent on the *E* field polarity. Theoretically, we address the role of the *E* field using first order perturbation theory to evaluate the free energy of the underlying phases. We show how an applied *E* field causes a relatively small shift of the SkL free energy that is nonetheless commensurate with the mean-field free energy difference between the competing skyrmion and conical phases, and thus dramatically controls the SkL phase stability. For the quantitative description of the experimental phase diagram, we develop a new approach for treating the fluctuative part of the free energy contributed by quasiparticle modes around *T*_*C*_. The inclusion of these modes proves pivotal for the correct evaluation of the free energy difference between the competing skyrmion and conical phases, both with and without *E* field, and thus represents an improved approach more generally for the calculation of the skyrmion phase diagram.

## Results

### Controlling skyrmion phase stability using electric fields

From recent bulk susceptibility *χ*(*H*) measurements of Cu_2_OSeO_3_^23^, it was suggested that skyrmions may be “created” or “annihilated” by applying a dc *E*-field in suitable parts of the temperature–magnetic field (*T*,*H*) phase diagram. In that study^23^ the skyrmion phase is identified by a small drop in *χ*(*H*), which serves as an indirect indication for the existence of the skyrmion phase.

We have used the tool of SANS to observe directly the microscopic skyrmionic magnetism in Cu_2_OSeO_3_ and its response to an applied dc *E*-field. In SANS the SkL phase is typically evidenced by a sixfold symmetric diffraction pattern, consistent with the so-called multi-*q* (triple-*q*) magnetic structure ansatz for the SkL described by three propagation *q*-vectors rotated by 120° with respect to each other (note that both ±**q** each give a SANS diffraction spot)^6,14,27^. In our SANS experiments we oriented the sample so that *E*||*H*|| [111], since according to previous bulk measurements^7^, the effect of the *E* field is expected to be maximal in this geometry. By measuring with the neutron beam also along [111], scattered intensity is only observed due to the SkL phase; for both the conical phase with *q*||*H* and the zero field helical phase with *q*||{100} type directions^6^, the expected scattering lies well out of the detector plane.

**Figure 1:**
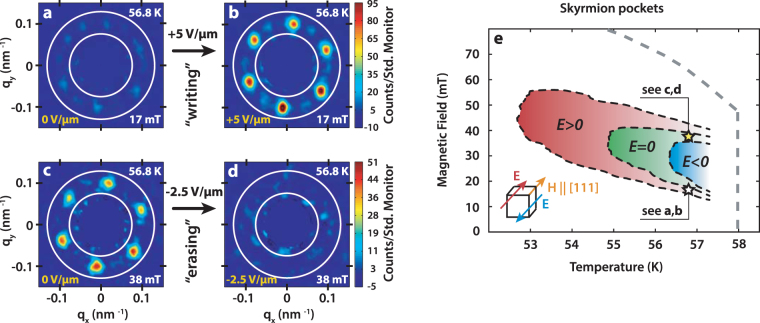
Skyrmion phase tuning by electric fields. (**a**–**d**) Representative SANS diffraction patterns obtained from the SkL phase at a temperature of 56.8 K, and various (*H*, *E*) conditions for $E\Vert H\Vert \mathrm{[111]}$. Here the [111] direction is into the page. Starting with only a weak intensity from the SkL at *T* = 56.8 K and *H* = 17 mT (a nearly conical state) (**a**), applying a positive *E* field enhances the SkL stability as evidenced by a significantly enhanced SkL intensity (**b**). Conversely, starting from a well-developed SkL state at *T* = 56.8 K and *H* = 38 mT (**c**), applying a negative *E* field erases the SkL (**d**). For the systematic analysis of the SANS data shown later in Figs 1(e) and 2(a–c), we evaluate the entire SANS intensity due to all SkL domains within the annular integration windows denoted by white rings in (**a**–**d**). This allows us to cater for scattering due to the major SkL domain (six strongest spots), and also any minority domains present that are signified by weaker diffraction spots in-between the strong ones. (**e**) shows the extent of the skyrmion phase pockets in the phase diagram for various *E* fields as determined by SANS. The skyrmion pocket doubles in temperature extent under a positive *E* field of +5.0 V/*μ*m, while shrinks to half its size at *E* = 0 under a negative *E* field of −2.5 V/*μ*m. The white and yellow stars on figure (**e**) mark experimental conditions of SANS patterns (**a**–**d**) on the phase diagram.

Figure 1 summarizes the direct *E*-field control of the SkL phase stability in Cu_2_OSeO_3_. Representative SANS data collected at constant *T* = 56.8 K, and at (*H*,*E*) coordinates selected to emphasise the *E* field effect, are shown in Fig. 1a–d. The initial states at T = 56.8 K were always prepared after zero-field cooling. By applying H = 17 mT at E = 0, the system the system is located on the border of the SkL phase and mostly in the conical phase, as evidenced by a weak SkL signal on the SANS detector (Fig. 1a). Without changing *T* or *H*, applying a positive *E* field of +5.0 V/*μ*m leads to the appearance of the characteristic 6-fold diffraction pattern in the SANS image (Fig. 1b), demonstrating the creation of a well-developed SkL. Conversely, starting from the SkL state at *H* = 0 38 mT and *E* = 0 (Fig. 1c), application of a negative electric field *E* = −2.5 V/*μ*m erases the SkL as evidenced by the disappearance of the SANS diffraction peaks (Fig. 1d).

Since in our experimental geometry it is only scattering from the SkL that contributes to the observed SANS intensity, we determined the extent of the SkL phase by evaluating the total intensity within the region of interest on the detector (the annular range within the white rings shown in Fig. 1a–d) as a function of *T*, *H*, and *E*. The resulting stability range of the skyrmion phase is shown in Fig. 1e for zero (green), positive (red) and negative (blue) *E* fields. We find that a positive *E* field of +5.0 V/*μ*m expands the skyrmion pocket so that it becomes twice larger (in temperature), while a negative *E* field of just *E* = −2.5 V/*μ*m shrinks the pocket by a factor of two. These SANS results provide microscopic experimental evidence for the electric field control of the skyrmion phase. Moreover, the results are quantitatively consistent with those of the indirect measurements reported in ref.^23^ after rescaling the data to take into account the difference between the ranges of applied electric field explored in the two studies.

### Optimum conditions for stabilising and destabilising the skyrmion phase

**Figure 2:**
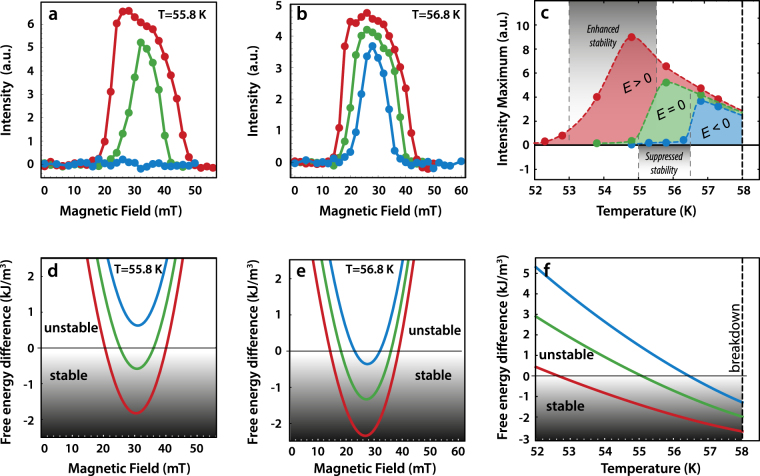
Skyrmion lattice stability in electric field: experiment and theory (**a,b**) *E*- and *H*-field dependence of the total scattered SANS intensity from SkLs in the sample at (**a**) *T* = 55.8 K, and (**b**) *T* = 56.8 K. The intensity evaluated is that observed within the annular sectors defined by white rings in Fig. 1a–d. (**c**) The *E*- and *T*-dependence of the peak SANS intensity in the *H*-scans like those shown in (**a,b**). In (**a**–**c**), symbols and lines in red, green and blue denote data obtained under *E* = +5kV/mm, *E* = 0 and *E* = −2.5 kV/mm, respectively. The dotted lines in (**c**) are guides for the eye. (**d,e**) The *H*-dependence of the calculated free energy difference between skyrmion and conical phases at (**d**) *T* = 55.8 K and (**e**) *T* = 56.8 K, for various *E*-fields. (**f**) The *T*-dependence of the minima in the calculated free energy differences like those shown in (**d,e**). In (**d**–**f**) calculations for *E* = +5 kV/mm, *E* = 0 kV/mm, *E* = −5 kV/mm are shown in red, green, blue, respectively (asymmetric ${{\rm{\ae }}}^{2}$ effects neglected). The skyrmion phase stability is preferred over the conical phase when the calculated free energy differences are negative.

Examining our SANS data more closely reveals the systematic manner by which applied *E* fields modify the SkL stability in the phase diagram. Figure 2a,b, show *H*-scans of the total scattered SANS intensity from the SkL for various *E* fields, and at *T* = 55.8 K and 56.8 K, respectively. At each *T*, the extent in *H* over which the SkL intensity is observed at *E* = 0 becomes enhanced under positive *E* fields, and suppressed under negative *E* fields. This demonstrates clearly the importance of the *E* field polarity on either enhancing or suppressing SkL stability. The effect is very pronounced as seen in Fig. 2a; at *T* = 55.8 K a negative *E* field can completely destabilise the SkL that is otherwise stable in the unperturbed state (*E* = 0).

Since the total scattered SANS intensity is indicative of both the population (relative to the conical phase) and quality of SkLs in the sample, the maximum intensity in the *H* scans like those shown in Fig. 2a,b is identified to represent the optimal SkL stability at each *E* and *T*. We present the *T*-dependence of this intensity peak at each *E* field in Fig. 2c, along with *T* windows identified as optimal for either enhancing or suppressing the SkL stability using the *E* field. Within the *T* window of 53–55.5 K, positive *E* fields enhance the SkL phase stability relative to the case at *E* = 0, while in the *T*-window of 55–56.5 K the SkL phase stability is readily suppressed by the negative *E* field relative to the *E* = 0 case. Positioning a sample at 55.5 K allows the demonstration of either a significant enhancement or suppression of the skyrmion phase at *E* = 0 by using *E*-fields of opposite polarity. In what follows, we develop a theory capable of explaining these observations quantitatively.

### Free energy in electric fields

The underlying mechanism for the *E* field driven enhancement or suppression of the skyrmion phase stability relies upon the magnetoelectric (ME) coupling in insulating Cu_2_OSeO_3_, which microscopically originates from the *d*-*p* hybridisation mechanism^27–31^. The emergent electric dipole moment **P** = *λ*(*S*_*y*_*S*_*z*_, *S*_*z*_*S*_*x*_, *S*_*x*_*S*_*y*_) is generated by the underlying spin structure **S**(***r***) = (*S*_*x*_, *S*_*y*_, *S*_*z*_), with the coupling between the magnetic and electric degrees of freedom described by a ME coupling parameter *λ* of relativistically small magnitude. Crucially, because the skyrmion phase now carries a non-vanishing electric-dipole moment, the ME coupling results in a **P**·**E** shift of the skyrmion free energy in *E*-field. This perturbation renormalises the elementary helices upon which the skyrmion phase is built, and slightly distorts the SkL^6,32^.

In this work, we apply the ME perturbation to the free energy described by an effective Ginsburg-Landau functional with Dzyaloshinski-Moriya interaction (DMI), and consider carefully the critical fluctuations that favour the skyrmion phase with respect to the competing conical phase (see Methods). Due to the relativistically small size of *λ*, the dimensionless *E*-field itself is rather small so that, ${\rm{\ae }}=\lambda E\mathrm{/4}D{k}_{0}\ll 1$, and we can build a perturbation theory in ${\rm{\ae }}$; for the modified free energy, neglecting all the terms of order ${{\rm{\ae }}}^{{\rm{2}}}$ and higher. Our finding is that perturbations of the fluctuative terms are important only at second order, while the mean-field energy already shifts in the first order due to direct ME and nonlinear contributions (see Methods). The corresponding shift in free energy of the skyrmion phase depends on the direction of *E*-field (see Fig. 2d–f), thus either enhancing (*E* > 0) or suppressing (*E* < 0) the skyrmion phase stability. While at first sight it can be surprising that the perturbation due to only a moderate applied *E*-field can play such a crucial role here, this is facilitated by the very close competition between the skyrmion and conical phases already in the mean-field.

### Calculation of the phase diagram

To calculate the response of the phase diagram to applied *E*-field, we use a new approach developed on the basis of effective models presented in refs^6,14,33^ (see Methods). Compared with these earlier studies, the new approach is self-consistent in the way that it reproduces the phase diagram, provides a deeper understanding of the role of quasiparticle modes near *T*_*C*_, and includes the path-integral approach presented previously for calculating the fluctuative free energy^14^ as a limiting case. We thus treat the first-order perturbation in *E* field on top of the mean-field solution, and add the fluctuative contributions that stabilise the SkL in the bulk. The main contribution of the *E* field here is captured by the shift of the mean-field free energy difference between the SkL and conical phases, while the fluctuative shift under voltage remains quadratically small.

Figure 2d,e each show calculations of the *H* and *E* field-dependent free energy difference between the skyrmion and conical phases, at *T* = 55.8 K and *T* = 56.8 K, respectively. In general, the calculations show the minima of the free energy difference curves deepen with increasingly positive *E* field. At *T* = 55.8 K changing between sufficiently negative and positive *E* fields can shift the curve so that the conical phase favoured for all *H* in a negative *E* field becomes unstable towards the skyrmion phase formation in the positive *E* field. This result is consistent with the experimental data shown in Fig. 2a. We also identify semi-quantitative agreement between these calculations and our experiments; the *H* location of the minima in the calculated free-energy difference curves [Fig. 2d,e] correspond well to the values of *H* where the peaks of SANS intensity are observed in the scans shown in Fig. 2a,b. In addition, we can link the calculated *T* and *E* field dependence of the free energy difference minima shown in Fig. 2f to our experimental data. The *T* s at which the free energy difference between the SkL and conical phases vanish for the theoretical *E* fields correspond to the *T* windows identified from the experimental data as optimal for either enhancing or suppressing SkL stability with *E* [Fig. 2c]. This correspondence between experiment and theory provides firm support for the validity of our theoretical treatment of the *E*-field effect on the SkL phase stability.

**Figure 3:**
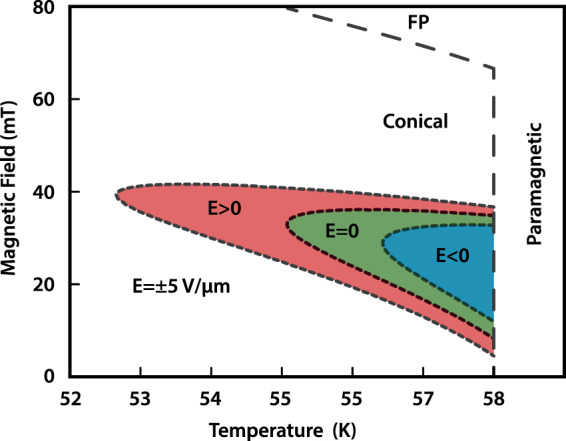
Calculation of the skyrmion phase in electric fields Calculated *H* and *T* extent of the skyrmion phase for *E* > 0 (red shading), *E* = 0 (green shading), *E* < 0 (blue shading) for the $E\Vert H\Vert \mathrm{[111]}$ geometry, and for the symmetric-response approach $({{\rm{\ae }}}^{1})$. The extent of each phase corresponds to the range over which the free energy of the skyrmion phase is less than that of the surrounding conical phase. Dashed lines denote phase boundaries, including the vertical breakdown regime at *T*_*C*_.

Independent of any applied *E* field, our theory provides a more general understanding of SkL stability on an intuitive, pictorial level: the critical fluctuations (waves) are superposed on top of the variationally minimised free energies. There are three critical modes ${\omega }_{{\bf{k}}}^{\mathrm{(0,1,2)}}$ around the mean-field (see Methods), with ${\omega }_{{\bf{k}}}^{\mathrm{(0)}}$ soft on the sphere $|{\bf{k}}|={k}_{0}$, which means that it costs very little energy to add many such fluctuations if they are coherent with the helix *k*_0_. Thus ${\omega }_{{{\bf{k}}}_{0}}^{\mathrm{(0)}}$ is the so-called “dangerous” mode since it results in a Van-Hove-like singularity at *T*_*C*_ and eventually breaks down the ordered phases into the disordered (paramagnetic) phase^33^. Below *T*_*C*_ the symmetry-breaking can be observed using SANS by either a six-fold pattern (SkL phase) or a two-fold pattern (helical or conical phases), both circumscribed on a sphere of radius $|{\bf{k}}|={k}_{0}$ in reciprocal space. Our calculation shows that the skyrmion phase is favoured because adding fluctuations generates more entropy in the skyrmion phase. This analysis also leads to a qualitative criterion for capturing the magnetic-field-independent breakdown of the ordered phases at *T*_*C*_ (see Methods). Asymptotically, the main contribution of the fluctuative free energy is given in the short-scale physics, where Cu_2_OSeO_3_ is “almost” a ferromagnet, thus reproducing the result of the path-integral approach^14^ as a limiting case. The model described here captures the qualitative physics of the system, as exemplified by the theoretical phase diagram shown in Fig. 3. To date, a quantitative theory for the skyrmion phase diagram under electric fields has been missing.

## Discussion

In some respects, the effect of *E* field observed here resembles that achieved due to applied uniaxial pressure^24,25^ since, the SkL phase stability can be either enhanced or suppressed by appropriate selection of the uniaxial stress direction relative to the direction of *H*. However, integrating the pressure effect on skyrmion stability into a technological setting is very challenging. In contrast, the *E* field is a versatile and reliable external parameter; providing an efficient control of both the skyrmion position^6,8,26^ and, as we show here the stability of the phase as a whole. Since the *E* field controlled expansion or contraction of the skyrmion phase can occur in general for an insulating ME skyrmion host with any *T*_*C*_, our findings are very attractive for applications at room *T*; for a device layer of thickness 100 nm the skyrmion phase in a sample can be entirely destabilised (erased) or restabilised (written) with less than 1 V, a voltage compatible with modern microelectronics.

The present study further lays both theoretical and experimental foundations for fully exploring alternative *H* and *E* field configurations, not only in reciprocal-space measurements like SANS, but also by real-space imaging techniques such as cryo-Lorentz transmission electron microscopy (LTEM) on technologically-relevant, nanometrically-thin specimens. Crucial next steps experimentally concerning the skyrmion writing and erasing includes exploring directly the expected *E* field-driven switching hysteresis between the competing conical and skyrmion states, and the associated volatility of remnant states in zero biasing E field. In addition, learning how E field influences out-of-equilibrium and metastable skyrmion configurations in confined geometries can provide progressive insights for assessing the merits of insulating skyrmions for practical uses.

In conclusion, we have demonstrated both theoretically and experimentally the mechanism by which a moderate electric field can either enhance or suppress skyrmion phase stability in the magnetoelectric chiral magnet Cu_2_OSeO_3_. In addition, we have provided the parameters by which our theoretical approach achieves quantitative agreement with experiment, and which can be extended towards describing the effect of *E* field on both stable and metastable skyrmion states, these being of paramount technological importance.

## Methods

### Small-angle neutron scattering (SANS)

For the SANS experiment, we used a single crystal crystal grown using chemical vapour transport^34^. The crystal with a *T*_*C*_ = 58 K was of mass 6 mg and volume 3.0 × 2.0 × 0.50 mm^3^ with the thinnest axis ||[111], and $[\bar{1}\bar{1}2]$ vertical. The sample was mounted onto a bespoke sample stick designed for applying dc *E*-fields^35^, and oriented with the orthogonal $[\bar{1}\bar{1}2]$ and $[1\bar{1}0]$ directions lying in the SANS scattering plane. For the SANS images shown in Fig. 1, the $[\bar{1}\bar{1}2]$ direction is aligned with the vertical axis, and the $[1\bar{1}0]$ direction aligned with the horizontal axis. In our experiments we achieved *E*-fields ranging from +5.0 kV/mm to −2.5 kV/mm. Evidence of electrical breakdown was detected for *E*-fields outside this range.

The sample was loaded into a horizontal field cryomagnet at the SANS-II beamline, SINQ, PSI. The magnetic field (*μ*_0_*H*) was applied parallel to both the [111] direction of the sample and the incident neutron beam to give the experimental geometry *E||μ*_0_*H||*[111]. In this geometry, the SANS signal is only detected from the skyrmion phase, which typically presents as a hexagonal scattering pattern with propagation vectors **q** ⊥*μ*_0_*H*. In this geometry, we avoid detecting any SANS signal due to either of the neighbouring helical (**q** ||{100}) or conical phases (**q** ||*μ*_0_*H*), since the propagation vectors of these phases lie well out of the SANS detector plane.

We used incident neutrons with a wavelength of 10.8 Å (Δ*λ*/*λ* = 10%). The scattered neutrons were detected using a position-sensitive multidetector. The SANS measurements were done by rotating (‘rocking’) the sample and cryomagnet ensemble over angles that brought the various SkL diffraction spots onto the Bragg condition at the detector. Data taken at 70 K in the paramagnetic state were used for background subtraction. Before starting each *μ*_0_*H*-scan, the sample was initially zero field-cooled from 70 K to a target temperature, with the *E*-field applied when thermal equilibrium was achieved. The *E*-field was maintained during the *μ*_0_*H*-scan. At each *T* we define the *μ*_0_*H* extent of the SkL phase as that over which SANS intensity is detected. We use this criterion to extract the parametric extent of the SkL phase for (*μ*_0_*H*,*T*,*E*) as shown in Figs 1 and 2. See Supplemental Material for more details.

### Mean-field free energy

The effective mean-field theory is based on the coarse-grained magnetisation approach $M({\bf{r}})={M}_{s}{\bf{S}}({\bf{r}})$ as described in^14^. One starts with the mean-field approach with free energy

1 $$F[{\bf{M}}]=\langle {{\rm{\Theta }}}_{T}\,{{\bf{M}}}^{2}+J{(\nabla {\bf{M}})}^{2}+D\,{\bf{M}}\cdot (\nabla \times {\bf{M}})+U{{\bf{M}}}^{4}-{\bf{H}}\cdot {\bf{M}}\rangle $$

with spatial average $\langle \mathrm{...}\rangle =\int \frac{dV}{V}\mathrm{...}$, and ${{\rm{\Theta }}}_{T}\propto \alpha (T-{T}_{C})$ near *T*_*C*_, *J* is the Heisenberg stiffness and *D* is DMI, *H* is the magnetic field, and the higher-order term *U* grants the formation of the crystalline phase^14^. In the mean-field, the interplay between Heisenberg and DMI energies determines the helical vector as *k*_0_ = *D*/2*J*. The long-range-ordered hexagonal skyrmion lattice is approximated as ${\bf{S}}({\bf{r}})\simeq {\bf{m}}+\mu {\sum }_{{{\bf{q}}}_{n}}{{\bf{S}}}_{{{\bf{q}}}_{n}}{e}^{i{{\bf{q}}}_{n}{\bf{r}}+i{\varphi }_{n}}+{\rm{c}}{\rm{.c}}{\rm{.}}$, where the summation runs over the magnetocrystalline wave vectors **q**_1_ + **q**_2_ + **q**_3_ = 0. In the mean-field, the skyrmion phase is slightly gapped with respect to the conical phase, however the two are closely competing. Further details of the mean-field theory described in ref.^14^.

### Perturbation theory in electric fields

The magneto-electric coupling in Cu_2_OSeO_3_ is relativistically small, so the perturbation parameter is ${\rm{\ae }}=\lambda E\mathrm{/4}D{k}_{0}\ll 1$. It is sufficient to use the first order perturbation theory on top of the non-perturbed free energy. We go to the rotated frame defined by the magnetic field direction along [111], and re-write the free energy. The first order perturbation theory gives eigenvectors:

2 $$\begin{array}{l}|{{\bf{S}}}_{{\bf{k}}}^{({\rm{\ae }})}\rangle =|{{\bf{S}}}_{{\bf{k}}}^{\mathrm{(0)}}\rangle +\sum _{n\ne 0}|{{\bf{S}}}_{{\bf{k}}}^{(n)}\rangle \frac{\langle {{\bf{S}}}_{{\bf{k}}}^{(n)}|{\hat{ {\mathcal H} }}^{({\rm{\ae }})}|{{\bf{S}}}_{{\bf{k}}}^{\mathrm{(0)}}\rangle }{{\varepsilon }_{{\bf{k}}}^{\mathrm{(0)}}-{\varepsilon }_{{\bf{k}}}^{(n)}}+{\mathscr{O}}({{\rm{\ae }}}^{2}),\end{array}$$

which are now the basis for constructing the distorted skyrmion lattice. For other (**H**, **E**)-field configurations, we re-do the calculations in the new rotated frames. See Supplemental Materials for further details.

### Fluctuation-induced phase stabilisation

We use a new approach, which captures as a limiting case the fluctuation free energy from^14^. The essential physics is captured already in Gaussian (noninteracting) fluctuations with free energy density

3 $$\begin{array}{l}\begin{array}{c}{F}_{{\rm{fluct}}}=\sum _{i}\sum _{{\bf{k}}}^{|{\bf{k}}| < {\rm{\Lambda }}}{\omega }_{{\bf{k}}}^{(i)}{f}_{{\bf{k}}}^{(i)}-T\,{{\rm{S}}}_{{\rm{fluct}}},\end{array}\end{array}$$

where Λ = 2*π*/*a* is the natural cut-off, ${f}_{{\bf{k}}}^{(i)}$ is the critical modes distribution, and the entropy of Gaussian fluctuations is

4 $$\begin{array}{l}\begin{array}{c}{{\rm{S}}}_{{\rm{fluct}}}=\sum _{i}\sum _{{\bf{k}}}^{|{\bf{k}}| < {\rm{\Lambda }}}\{(1+{f}_{{\bf{k}}}^{(i)})\mathrm{ln}(1+{f}_{{\bf{k}}}^{(i)})-{f}_{{\bf{k}}}^{(i)}\,\mathrm{ln}\,{f}_{{\bf{k}}}^{(i)}\}\end{array}\end{array}$$

in the case of bosons. Fluctuations around mean-field are described by the generalised susceptibility ${\chi }_{ij}^{-1}({\bf{r}},{\bf{r}}\text{'})=\frac{1}{T}\frac{{\delta }^{2}F}{\delta {M}_{i}({\bf{r}})\delta {M}_{j}({\bf{r}}\text{'})}$, giving rise to several collective modes (See Supplemental Material). On the local scale $(k\gg J/D)$, the chiral magnet is reminiscent of a ferromagnet, so the modes behave asymptotically *ω*_**k**_ ∝ *k*^2^ for large *k*, thus asymptotically ${F}_{{\rm{fluct}}}\simeq \,\mathrm{log}\,\beta {\omega }_{{\bf{k}}}\propto \,\mathrm{log}\,{k}^{2}$, which covers the model of ref.^14^. The main contribution to (5) is given by the short length-scale (“ferromagnetic”) physics,

5 $$\begin{array}{l}{\rm{\Delta }}{F}_{{\rm{fluct}}}\simeq \frac{10U}{\pi Da}\langle {{\bf{S}}}_{{\rm{SkL}}}^{2}-{{\bf{S}}}_{{\rm{con}}}^{2}\rangle T,\end{array}$$

The electric field also slightly affects the fluctuative energy, because it modifies the correlation length near *T*_*C*_ and so renormalises *J*_eff_, which is neglected here as a higher-order $({{\rm{\ae }}}^{2})$ effect. See [Supplemental Material] for further details.

### Parameters of the effective model

For our numerical calculations we use *T*_*C*_ = 58 K, which approximately sets the Heisenberg stiffness as *J* = 4.85 × 10^−23^ Jm/A^2^. From the SANS measurement we establish directly the modulation period of 60 nm, which estimatively differs by a few percents from the mean-field value 2*π*/*k*_0_, because the mean-field ordering vector *k*_0_ = *D*/2*J* is slightly renormalised by the fluctuations near *T*_*C*_. This sets the “bare” DM interaction entering (1) as *D* = −9.85 × 10^−15^ J/A^2^. The lattice parameter is *a* = 8.91 × 10^−10^ m, which gives the natural cutoff Λ = 2*π*/*a *≈ 70*k*_0_. The saturation magnetization in Cu_2_OSeO_3_ is *M*_*s*_ = 1.11 × 10^5^ A/m and scales with temperature as ${M}_{s}(T)={M}_{s}{(1-{(T/{T}_{C})}^{{\alpha }_{1}})}^{{\alpha }_{2}}$, with *α*_1_ = 1.95 and *α*_2_ = 0.393^36^. We choose the nonlinear coupling responsible for SkL formation *U* = 6.2 × 10^−6^ Jm^−1^ A^−2^ and Landau parameter ${\alpha }_{T}={\theta }_{T}/J{k}_{0}^{2}(T-{T}_{C}^{{\rm{m}}{\rm{.f}}{\rm{.}}})=3.5\,{{\rm{K}}}^{-1}$. For the qualitative phase diagram shown in Fig. 3, we use a symmetric-response model $({\text{ae}}^{1})$, for which the best fit to SANS data is for ${\text{ae}}^{1}=0.02$, which corresponds here to *E* = ±5 × 10^6^ V/m coupled with *λ*/*Dk*_0_ = 9.23 × 10^−3^ *μ*m/V to the underlying spin structure through ME mechanism.

## Electronic supplementary material

Supplementary information
