# Supplementary material for: Direct electric field control of the skyrmion phase in a magnetoelectric insulator
Source: Sci Rep. 2018 Jul 11;8:10466. doi: 10.1038/s41598-018-27882-4 (PMC6041276; doi:10.1038/s41598-018-27882-4)
Supplement: Supplementary file 1 — Supplementary information [file 41598_2018_27882_MOESM1_ESM.pdf]

Supporting Information for "Direct electric field control of the skyrmion phase in a magnetoelectric insulator"

A. J. Kruchkov,[1,2] J. S. White,[3] M. Bartkowiak,[4] I. Živković,[2] A. Magrez,[5] and H. M. Rønnow[2]

[1] *Department of Physics, Harvard University, Cambridge, MA 02138, USA*
[2] *Laboratory for Quantum Magnetism (LQM), Ecole Polytechnique Fédérale de Lausanne (EPFL), CH-1015 Lausanne, Switzerland*
[3] *Laboratory for Neutron Scattering (LNS), Paul Scherrer Institut (PSI), CH-5232 Villigen, Switzerland*
[4] *Laboratory for Scientific Developments and Novel Materials (LDM), Paul Scherrer Institut (PSI), CH-5232 Villigen, Switzerland*
[5] *Crystal Growth Facility, Ecole Polytechnique Fédérale de Lausanne (EPFL), CH-1015 Lausanne, Switzerland*

(Dated: May 2, 2018)

A. Effective model in a coarse-grained approximation

The Skyrmion Lattice (SkL) is a long-range-order spin configuration which can be visualized as a hexagonal lattice of vortices. Experimentally, the hallmark of the SkL phase is the appearance of a six-fold symmetric reflection pattern in reciprocal space, with each of the three associated propagation vectors mutually rotated with respect to one another by $2\pi/3$ (see e.g. Refs. [1, 3] for typical small-angle neutron scattering (SANS) patterns from the SkL). In this study, we describe the SkL by a coarse-grained local magnetization vector $\mathbf{S}(\mathbf{r})$, which can be built from three propagation, or $\mathbf{Q}$-vectors. To good accuracy the SkL phase can be approximated by the multispiral spin structure [3]

$$\mathbf{S}(\mathbf{r}) = \mathbf{m} + \mu \sum_{\mathbf{Q}_n} \mathbf{S}_{\mathbf{Q}_n} e^{i\mathbf{Q}_n \mathbf{r} + i\varphi_n} + \mathbf{S}^*_{\mathbf{Q}_n} e^{-i\mathbf{Q}_n \mathbf{r} - i\varphi_n}, \quad (1)$$

where $\mathbf{m} \equiv \langle \mathbf{S}(\mathbf{r}) \rangle$ is a uniform magnetization, with (spatial) average defined as $\langle ... \rangle = \int \frac{dV}{V} (...)$ throughout the study, and μ is the weight of the SkL modulation. The sum in (1) runs over the "3Q-structure", and the relative phases φ_n in (1) are important for minimization of the SkL energy. The expectation of the energy density in the coarse-grained model is obtained by calculating the spatial average $\langle \mathcal{H} \rangle$ with spin function

$$\mathcal{H} = \mathcal{H}_{JDh} + \mathcal{H}_A + \mathcal{H}_{æ}, \quad (2)$$

where the helimagnetic term

$$\mathcal{H}_{JDh} = J(\nabla \mathbf{S})^2 + D\mathbf{S} \cdot (\nabla \times \mathbf{S}) - \mathbf{h} \cdot \mathbf{S} \quad (3)$$

takes into account the Heisenberg interaction (J), the Dzyaloshinskii-Moriya interaction (D) and the Zeeman coupling to the external magnetic field $\mathbf{h} = \mathbf{H}/M_s$, where M_s is the saturation magnetization. In what follows we put $M_s = 1$ and $a = 1$, but restore them at the very end of the calculation.

In this study, we consider the fourth-order anisotropy as it represents the essential physics of the problem by stabilizing the SkL phase.[1, 3] The symmetry of Cu_2OSeO_3 is described by the $P2_13$ space group, which allows a fourth-order magneto-crystalline anisotropy of the form $A_1(S_x^4 + S_y^4 + S_z^4) + A_2(S_x^2 S_y^2 + S_y^2 S_z^2 + S_z^2 S_x^2)$. Proceeding via the unitary parametrization $\mathbf{S}/|\mathbf{S}| = (\sin\theta\cos\psi, \sin\theta\sin\psi, \cos\theta)$, one obtains $S_x^2 S_y^2 + S_y^2 S_z^2 + S_z^2 S_x^2 = -\frac{1}{2}(S_x^4 + S_y^4 + S_z^4) + \frac{1}{2}$, and we thus have:

$$\mathcal{H}_A = A(S_x^4 + S_y^4 + S_z^4) + U\mathbf{S}^4. \quad (4)$$

with $A = A_1 - A_2/2$ and $U = A_2/2$. Thus, there could be two distinct situations, first $A_1 \gg A_2$, for which the anisotropy of the bulk is important, and second, the case with $A_1 \sim A_2/2$, where the role of anisotropy is reduced to providing mode-mode coupling $U \sim A_1, A_2$.

Finally, we make a remark on the importance of the anisotropy terms. The appearance of the six-hold set $\{\mathbf{Q}_1, \text{-}\mathbf{Q}_3, \mathbf{Q}_2, \text{-}\mathbf{Q}_1, \mathbf{Q}_3, \text{-}\mathbf{Q}_2\}$ of helices phased in such a way as to form the two-dimensional skyrmion crystalline can occur only as a consequence of higher-order energy terms than those represented in the model. A third-order term like $\mathbf{S}^3$ is forbidden however by a magnetic field promoted symmetry. Therefore, an anisotropy of at least fourth order can be considered as the source of the skyrmion lattice order parameter (1) in bulk chiral ferromagnets. [1] Indeed, we can consider the fourth order anisotropy which - fully or partially - contains the term $\mathbf{S}^4(\mathbf{r}) = S_x^4 + S_y^4 + S_z^4 + 2(S_x^2 S_y^2 + S_y^2 S_z^2 + S_z^2 S_x^2)$. After decoupling the uniform component, $\mathbf{S}(\mathbf{r}) = \langle \mathbf{S} \rangle + \mathbf{s}(\mathbf{r})$, the expansion will contain the cubic term

$$\mathbf{S}^4(\mathbf{r}) = [\mathbf{s}(\mathbf{r}) + \langle \mathbf{S}(\mathbf{r}) \rangle]^4 = ... + 4\mathbf{s}^2(\mathbf{r})\, \mathbf{s}(\mathbf{r}) \cdot \langle \mathbf{S}(\mathbf{r}) \rangle + ..., \quad (5)$$

which thus generates a third-order term with the momentum-conserving condition $\mathbf{Q}_1 + \mathbf{Q}_2 + \mathbf{Q}_3 = 0$,

[1] In metallic chiral magnets such as MnSi, the $\mathbf{S}^4$ term may appear as the result of conductive mode-mode interactions [3].

$$\langle \mathbf{s}^2(\mathbf{r})\,\mathbf{s}(\mathbf{r})\rangle = \sum_{\mathbf{Q}_i} (\mathbf{S}_{\mathbf{Q}_1}\cdot\mathbf{S}_{\mathbf{Q}_2})\,\mathbf{S}_{\mathbf{Q}_3}\,\delta(\mathbf{Q}_1+\mathbf{Q}_2+\mathbf{Q}_3). \tag{6}$$

Thus one can show that the solution for the SkL phase can be expressed through the three in-plane $\mathbf{Q}$ vectors, equirotated by $2\pi/3$, of arbitrary rotation angle ϕ in the plane. The multispiral SkL phase is expected to minimize the terms that include the form of (6) after an appropriate choice of variational order parameter components.

We next note that an effective description of the magnetic phase diagram in bulk skyrmion materials is already possible within the $U\mathbf{S}^4$ formalism. The remarkable universality of the phase diagram between different materials, and qualitatively invariant constitution of the phase diagram under sample rotation, leads us to conjecture that the rotationally-invariant $U\mathbf{S}^4$ term is responsible for most of the qualitative physics related with SkLs in bulk. Moreover, the observed *Brazovskii sphere* of critical fluctuations at T_C [2] indicate that the fluctuation-induced phase transition in these systems is of isotropic nature, and so the further order anisotropies (containing spatial derivatives) can also be neglected. Therefore we consider the effective energy function as

$$\mathcal{H}_{JDh} = J(\nabla\mathbf{S})^2 + D\,\mathbf{S}\cdot(\nabla\times\mathbf{S}) + U\mathbf{S}^4 - \mathbf{h}\cdot\mathbf{S}. \tag{7}$$

First, we discuss the chiral physics of the minimal model

$$W_0 = \langle J\,[\nabla\mathbf{S}(\mathbf{r})]^2 + D\,\mathbf{S}(\mathbf{r})\cdot[\nabla\times\mathbf{S}(\mathbf{r})]\rangle = \sum_{\mathbf{k}} \mathbf{S}^\dagger_{\mathbf{k}}\hat{\mathcal{H}}_0\mathbf{S}_{\mathbf{k}}, \tag{8}$$

Using the spin representation $\mathbf{S}_{\mathbf{k}} = (S^x_{\mathbf{k}}, S^y_{\mathbf{k}}, S^z_{\mathbf{k}})^T$, $\hat{\mathcal{H}}_0$ is written in as a matrix operator:

$$\hat{\mathcal{H}}_0 = \begin{pmatrix} Jk^2 & \text{-}iDk_z & iDk_y \\ iDk_z & Jk^2 & \text{-}iDk_x \\ \text{-}iDk_y & iDk_x & Jk^2 \end{pmatrix}. \tag{9}$$

The $\mathbf{Q}$-vectors of the SkL lie in the plane which is perpendicular to the magnetic field $\mathbf{h}$. Consequently, each of the six helices of the skyrmion lattice is parametrized as $\mathbf{k} = (k_{x'}, k_{y'}, 0)$ in the rotated frame, and the problem is effectively two-dimensional. The energy matrix ("hamiltonian") (9) in the rotated spin frame (see further) is

$$\hat{\mathcal{H}}_0 = \begin{pmatrix} Jk^2 & 0 & iDk_{y'} \\ 0 & Jk^2 & \text{-}iDk_{x'} \\ \text{-}iDk_{y'} & iDk_{x'} & Jk^2 \end{pmatrix}, \tag{10}$$

and is diagonalized on the eigenstates

$$|\mathbf{S}^{(0)}_{\mathbf{k}}\rangle = \frac{1}{\sqrt{2}}\left(\text{-}i\hat{k}_{y'}, i\hat{k}_{x'}, 1\right)^{\mathsf{T}}, \tag{11}$$

$$|\mathbf{S}^{(1)}_{\mathbf{k}}\rangle = \left(\hat{k}_{x'}, \hat{k}_{y'}, 0\right)^{\mathsf{T}}, \tag{12}$$

$$|\mathbf{S}^{(2)}_{\mathbf{k}}\rangle = \frac{1}{\sqrt{2}}\left(i\hat{k}_{y'}, \text{-}i\hat{k}_{x'}, 1\right)^{\mathsf{T}}, \tag{13}$$

where $\hat{k}_{x',y',z'} = k_{x',y',z'}/|\mathbf{k}|$, and we have introduced "bra" and "ket" notation as shortcuts for writing the perturbation formulas of the matrix mechanics [4] in a familiar way. We denote for brevity $\langle\mathbf{S}_{\mathbf{k}}|...|\mathbf{S}_{\mathbf{k}}\rangle = \sum_{\mathbf{k}}\mathbf{S}^\dagger_{\mathbf{k}}...\mathbf{S}_{\mathbf{k}}$, which is just a Fourier-transform of the corresponding spatial averaging $\langle ...\rangle$ as in Eq.(8).

The spectrum of the matrix operator $\hat{\mathcal{H}}_0$ consists of three equidistant energy solutions with separation in the energy of $\pm Dk$,

$$\varepsilon^{(0)}_{\mathbf{k}} = Jk^2 - Dk, \quad \varepsilon^{(1)}_{\mathbf{k}} = Jk^2, \quad \varepsilon^{(2)}_{\mathbf{k}} = Jk^2 + Dk. \tag{14}$$

For positive J, D the lowest-energy state is $|\mathbf{S}^{(0)}_{\mathbf{k}}\rangle$, which corresponds to the eigenvalue $\varepsilon^{(0)}_{\mathbf{k}}$. The modulation vector k_0 is determined by further minimization of the energy, $\partial\varepsilon^{(0)}_{\mathbf{k}}/\partial k = 0$, which gives $k_0 = D/2J$. Thus for further calculations we simply have

$$\varepsilon^{(0)}_{\mathbf{k}_0} = -Dk_0/2, \quad \varepsilon^{(1)}_{\mathbf{k}_0} = Dk_0/2, \quad \varepsilon^{(2)}_{\mathbf{k}_0} = 3Dk_0/2. \tag{15}$$

As a remark, it is usually convenient to measure the energy of the system in units of Dk_0.

To capture universal physics of the skyrmion-hosting phase diagram, we consider the effective $\mathbf{S}^4$ model, and construct a Landau-Ginzburg-like free energy of form,

$$F_0[\mathbf{M}(\mathbf{r})]/VM_s^2 = \langle\alpha_T\mathbf{S}^2 + J(\nabla\mathbf{S})^2 + D\,\mathbf{S}\cdot(\nabla\times\mathbf{S}) - \mathbf{h}\cdot\mathbf{S} + U\mathbf{S}^4\rangle. \tag{16}$$

In what follows, we first treat the mean-field free energy, and then calculate fluctuative corrections based on a calculation of the quasiparticle free energy. To take into account the electric (E) field control of the skyrmion phase stability, we use perturbation theory as described below. We show (see also further) that because of the relatively small size of the applied E fields, the important physics is already covered by the first-order (antisymmetric) shift of skyrmion phase free energies. We thus neglect all the next-order effects, including any E field-induced perturbation of the fluctuative part of the free energy.

The magneto-electric coupling in Cu_2OSeO_3 arises due to the p-d hybridization mechanism(see Refs. [5–9]), which gives rise to an electric dipole moment $\mathbf{P} = \alpha_\lambda(S_yS_z, S_zS_x, S_xS_y)$, i.e. an *electric dipole moment*

coupled directly to the spin variables S_x, S_y, S_z. Therefore, in external E field the ordered phase is perturbed by a dipole energy $-\mathbf{P}\cdot\mathbf{E}$, or

$$\mathcal{H}_{æ} = \alpha E_x S_y S_z + \text{cyclic permutations}, \quad (17)$$

where $\mathbf{E} = (E_x, E_y, E_z)$ is the external E field, and for simplicity we absorbed the minus sign into $\alpha = -\alpha_\lambda$.

We consider now explicitly the field geometry $E||H||[1\,1\,1]$ consistent with out experiments. The rotated spin frame is given by a unitary linear transform

$$\mathbf{S_r} = \mathcal{R}_{[111]}\,\mathbf{S_{r'}}, \qquad \mathcal{R}_{[111]} = \begin{pmatrix} -\frac{1}{\sqrt{2}} & -\frac{1}{\sqrt{6}} & \frac{1}{\sqrt{3}} \\ \frac{1}{\sqrt{2}} & -\frac{1}{\sqrt{6}} & \frac{1}{\sqrt{3}} \\ 0 & \sqrt{\frac{2}{3}} & \frac{1}{\sqrt{3}} \end{pmatrix}, \quad (18)$$

for which the ME coupling reads

$$\mathcal{H}_{æ}/Dk_0 = 2æ \begin{pmatrix} -1 & 0 & 0 \\ 0 & -1 & 0 \\ 0 & 0 & 2 \end{pmatrix}, \quad (19)$$

with

$$æ = \frac{\alpha E}{4Dk_0}, \qquad \mathbf{E} = (E, E, E), \qquad E = \frac{|\mathbf{E}|}{\sqrt{3}}. \quad (20)$$

In this study we are interested primarily in first order perturbation theory, thus only the higher-order terms contribute. We thus use

$$|\mathbf{S}_\mathbf{k}^{(æ^1)}\rangle = |\mathbf{S}_\mathbf{k}^{(0)}\rangle + \sum_{n\neq 0} |\mathbf{S}_\mathbf{k}^{(n)}\rangle \frac{\langle \mathbf{S}_\mathbf{k}^{(n)}|\hat{\mathcal{H}}_{æ}|\mathbf{S}_\mathbf{k}^{(0)}\rangle}{\varepsilon_\mathbf{k}^{(0)} - \varepsilon_\mathbf{k}^{(n)}} + \mathcal{O}(æ^2). \quad (21)$$

where $\varepsilon_\mathbf{k}^{(n)}$ are given by Eq.(15) and $|\mathbf{S}_\mathbf{k}^{(n)}\rangle$, $n = 0, 1, 2$, are the eigenstates of $\mathcal{H}_0$ as given by Eqs.(11) -(12). The direct calculation for perturbation (19) is

$$|\mathbf{S}_\mathbf{k}^{(æ^1)}\rangle = |\mathbf{S}_\mathbf{k}^{(0)}\rangle - æ\,|\mathbf{F}_\mathbf{k}\rangle, \quad (22)$$

with the elastic vector of form

$$|\mathbf{F}_\mathbf{k}\rangle = \frac{3}{2\sqrt{2}} \begin{pmatrix} -i\sin\phi \\ +i\cos\phi \\ -1 \end{pmatrix} = \frac{3}{2}|\mathbf{S}_\mathbf{k}^{(0)}\rangle - 2|\mathbf{S}_0^{(0)}\rangle, \quad (23)$$

where $|\mathbf{S}_\mathbf{k}^{(0)}\rangle$ is given by (11) and $|\mathbf{S}_0^{(0)}\rangle \equiv (0,0,1)^T$ is a field-polarized $k = 0$ component. Thus we have simply

$$|\mathbf{S}_\mathbf{k}^{(æ^1)}\rangle = \left(1 + \frac{3}{2}æ\right)|\mathbf{S}_\mathbf{k}^{(0)}\rangle - 2æ\,|\mathbf{S}_0^{(0)}\rangle, \quad (24)$$

and in the skyrmion phase we have

$$\begin{aligned}
\langle \mathbf{S}^4(\mathbf{r})\rangle &= m^4 + \\
&+ 2m^2\mu^2 \sum_{\mathbf{k}_1,\mathbf{k}_2}^{\{\mathbf{q}_1\ldots\mathbf{q}_6\}} \left(S^x_{\mathbf{k}_1}S^x_{\mathbf{k}_2} + S^y_{\mathbf{k}_1}S^y_{\mathbf{k}_2} + 3S^z_{\mathbf{k}_1}S^z_{\mathbf{k}_2}\right)\delta(\mathbf{k}_1+\mathbf{k}_2) \\
&+ 4m\mu^3 \sum_{\mathbf{k}_1,\mathbf{k}_2,\mathbf{k}_3}^{\{\mathbf{q}_1\ldots\mathbf{q}_6\}} \left(S^z_{\mathbf{k}_1}S^x_{\mathbf{k}_2}S^x_{\mathbf{k}_3} + S^z_{\mathbf{k}_1}S^y_{\mathbf{k}_2}S^y_{\mathbf{k}_3} + S^z_{\mathbf{k}_1}S^z_{\mathbf{k}_2}S^z_{\mathbf{k}_3}\right) \\
&\times e^{i(\varphi_{\mathbf{k}_1}+\varphi_{\mathbf{k}_2}+\varphi_{\mathbf{k}_3})}\,\delta(\mathbf{k}_1+\mathbf{k}_2+\mathbf{k}_3) \\
&+ \mu^4 \sum_{\mathbf{k}_1,\mathbf{k}_2,\mathbf{k}_3,\mathbf{k}_4}^{\{\mathbf{q}_1\ldots\mathbf{q}_6\}} \left(S^x_{\mathbf{k}_1}S^x_{\mathbf{k}_2}S^x_{\mathbf{k}_3}S^x_{\mathbf{k}_4} + S^y_{\mathbf{k}_1}S^y_{\mathbf{k}_2}S^y_{\mathbf{k}_3}S^y_{\mathbf{k}_4}\right. \\
&+ S^z_{\mathbf{k}_1}S^z_{\mathbf{k}_2}S^z_{\mathbf{k}_3}S^z_{\mathbf{k}_3} + 2S^x_{\mathbf{k}_1}S^x_{\mathbf{k}_2}S^y_{\mathbf{k}_3}S^y_{\mathbf{k}_4} + 2S^y_{\mathbf{k}_1}S^y_{\mathbf{k}_2}S^z_{\mathbf{k}_3}S^z_{\mathbf{k}_4} \\
&\left. + 2S^x_{\mathbf{k}_1}S^x_{\mathbf{k}_2}S^z_{\mathbf{k}_3}S^z_{\mathbf{k}_4}\right)\delta(\mathbf{k}_1+\mathbf{k}_2+\mathbf{k}_3+\mathbf{k}_4), \quad (25)
\end{aligned}$$

where each $\mathbf{k}_i$ runs through $\{\mathbf{q}_1, \mathbf{q}_2, \mathbf{q}_3, \mathbf{q}_4, \mathbf{q}_5, \mathbf{q}_6\} = \{\pm\mathbf{Q}_1, \pm\mathbf{Q}_2, \pm\mathbf{Q}_3\}$. The direct substitution of (24) gives

$$\begin{aligned}
W^{(U)}_{SkL} = \langle U\,\mathbf{S}^4(\mathbf{r})\rangle_{æ} &= U\left(m^4 + 51\mu^4 + 24m^2\mu^2 - 18\sqrt{2}m\mu^3\right) \\
&- 9U\mu^2\left(6\mu^2 + 4m^2 - 5\sqrt{2}\mu m\right)æ. \quad (26)
\end{aligned}$$

For comparison, in the conical phase we only have

$$\begin{aligned}
\langle \mathbf{S}^4(\mathbf{r})\rangle &= m^4 + \quad (27) \\
&+ 2m^2\mu^2 \sum_{\mathbf{k}_1}^{\pm\mathbf{Q}}\sum_{\mathbf{k}_2}^{\pm\mathbf{Q}} \left(S^x_{\mathbf{k}_1}S^x_{\mathbf{k}_2} + S^y_{\mathbf{k}_1}S^y_{\mathbf{k}_2} + S^z_{\mathbf{k}_1}S^z_{\mathbf{k}_2}\right)\delta(\mathbf{k}_1+\mathbf{k}_2) \\
&+ \mu^4 \sum_{\mathbf{k}_1}^{\pm\mathbf{Q}}\sum_{\mathbf{k}_2}^{\pm\mathbf{Q}}\sum_{\mathbf{k}_3}^{\pm\mathbf{Q}}\sum_{\mathbf{k}_4}^{\pm\mathbf{Q}} \left(S^x_{\mathbf{k}_1}S^x_{\mathbf{k}_2}S^x_{\mathbf{k}_3}S^x_{\mathbf{k}_4} + S^y_{\mathbf{k}_1}S^y_{\mathbf{k}_2}S^y_{\mathbf{k}_3}S^y_{\mathbf{k}_4}\right. \\
&+ S^z_{\mathbf{k}_1}S^z_{\mathbf{k}_2}S^z_{\mathbf{k}_3}S^z_{\mathbf{k}_3} + 2S^x_{\mathbf{k}_1}S^x_{\mathbf{k}_2}S^y_{\mathbf{k}_3}S^y_{\mathbf{k}_4} + 2S^y_{\mathbf{k}_1}S^y_{\mathbf{k}_2}S^z_{\mathbf{k}_3}S^z_{\mathbf{k}_4} \\
&\left. + 2S^x_{\mathbf{k}_1}S^x_{\mathbf{k}_2}S^z_{\mathbf{k}_3}S^z_{\mathbf{k}_4}\right)\delta(\mathbf{k}_1+\mathbf{k}_2+\mathbf{k}_3+\mathbf{k}_4). \quad (28)
\end{aligned}$$

because the cubic term is always zero, and also $\langle \mathbf{m}\cdot\mathbf{s}\rangle = 0$ which results in only s_z^2 instead of $3s_z^2$ in the skyrmion phase. By repeating the perturbation approach (21) for the conical phase, a calculation for $E||[1\,1\,1]$ shows that the conical vectors remain unperturbed. Therefore for this field configuration, we have

$$W^{(U)}_{\text{con}} = \langle U\,\mathbf{S}^4(\mathbf{r})\rangle_{æ} = U\left(m^2 + 2\mu^2\right)^2. \quad (29)$$

The result is that in the main approximation, the conical phase for $E||H||[1\,1\,1]$ is not sensitive to E field, thus allowing the observation of a pure effect of E field on the SkL. This result is used for planning studies described in the next section. Note however that the conical vectors will be indeed perturbed for $H||[1\,\bar{1}\,0]$, though not

crucially.[2]

The present model thus describes a shift of the mean-field energy of the SkL, which is either positive or negative depending on the direction of the E field. In a particular situation when the first order terms may vanish, the energy shift is of the same sign for both positive and negative voltage polarities.

B. Susceptibility tensor and mean-field fluctuation spectrum

The noninteracting (Gaussian) critical fluctuations around the mean-field solution are described by the generalized susceptibility tensor $\chi_{ij}(\mathbf{r}, \mathbf{r}')$ defined as

$$\chi^{-1}_{\alpha\beta}(\mathbf{r}, \mathbf{r}') = \frac{1}{T} \frac{\delta^2 F}{\delta M^\alpha(\mathbf{r})\, \delta M^\beta(\mathbf{r}')} \tag{30}$$

A direct calculation gives

$$T\chi^{-1}_{\alpha\beta}(\mathbf{r}, \mathbf{r}') = \delta(\mathbf{r} - \mathbf{r}') \left[(\alpha_T - J\nabla^2)\delta_{\alpha\beta} - D\, \varepsilon_{\alpha\beta\gamma}\, \partial_\gamma \right. \\ \left. + \, 4U S^\alpha(\mathbf{r}) S^\alpha(\mathbf{r}) \delta_{\alpha\beta} + 8U S^\alpha(\mathbf{r}) S^\beta(\mathbf{r}) \right] . \tag{31}$$

The Fourier-transformed susceptibility reads

$$T\chi^{-1}_{\alpha\beta}(\mathbf{k}, \mathbf{k}') = \Theta_{\alpha\beta}(\mathbf{k}) \delta_{\mathbf{k}\,\mathbf{k}'} \tag{32}$$

$$+4U \sum_{\mathbf{k}''} \left(\delta_{\alpha\beta}\, S^\alpha_{-\mathbf{k}''}\, S^\alpha_{\mathbf{k}-\mathbf{k}'+\mathbf{k}''} + 2 S^\alpha_{-\mathbf{k}''}\, S^\beta_{\mathbf{k}-\mathbf{k}'+\mathbf{k}''} \right) , \tag{33}$$

with $\Theta_{\alpha\beta}(\mathbf{k}) = (Jk^2 + \alpha_T)\delta_{\alpha\beta} - iD\varepsilon_{\alpha\beta\gamma} k_\gamma$. The spectrum of Gaussian fluctuations is thus given by the poles of the susceptibility $\chi_{\alpha\beta}(\mathbf{k}, \mathbf{k}')$, and the eigenvalue equation reads

$$T \sum_{\mathbf{k}} \chi^{-1}_{ij}(\mathbf{k}, \mathbf{k}') \nu_i(-\mathbf{k}) = \omega_{\mathbf{k}}\, \nu_i(\mathbf{k}), \tag{34}$$

where $\nu_i(-\mathbf{k})$ are eigenvectors. It is fruitful to firstly consider the mean-field fluctuation spectrum in the paramagnetic phase ($\langle M_i \rangle = 0$, $\langle M_i^2 \rangle = 0$) close to the critical point. The corresponding spectrum has three branches[3]

$$\omega^{(0)}_{\mathbf{k}} = J(k - k_0)^2 + \delta(T), \\ \omega^{(1)}_{\mathbf{k}} = J(k^2 + k_0^2) + \delta(T), \\ \omega^{(2)}_{\mathbf{k}} = J(k + k_0)^2 + \delta(T), \tag{35}$$

where $\delta(T)$ is the detuning parameter, see Ref[2] for more details. The "dangerous" mode is $\omega^{(0)}_{\mathbf{k}}$ which is soft at $k_0 = D/J$ at T_c where $\delta(T_c) = 0$. This causes fluctuations on the 3D Brazovskii sphere of radius $\mathbf{k} = k_0$, and which leads to field amplitude-squared singularity observed by neutron scattering as a uniform sphere of scattering at $T = T_c$. This symmetry on a 3D sphere $\mathbf{k} = k_0$ is broken further below the critical point; either into 3Q-helices defining a plane (the skyrmion phase: $\mathbf{k}_1 + \mathbf{k}_2 + \mathbf{k}_3 = 0$, i.e. the sixfold scattering pattern with $k = k_0$), or a 1-helix defining a line (helical or conical phase, two-fold scattering pattern). The dispersions $\omega^{(0)}_{\mathbf{k}}$, $\omega^{(1)}_{\mathbf{k}}$, $\omega^{(2)}_{\mathbf{k}}$ also changed below T_C since the average magnetization is not vanishing, and thus anisotropy-induced terms (e.g. those with U in (32)) come into a play. However, the limiting case $k \to \infty$ for all the branches is $\omega_{\mathbf{k}} \sim Jk^2$, which means physically that on the short length scales the chiral magnet is ferromagnetic in nature.

C. Fluctuative contribution to self-energy

The surprising finding of the theory reported in Ref. 3 is that the main contribution to the fluctuative free energy is given by the short scale fluctuations, as further verified numerically [3]. Here we consider a different way of deriving this result, which is more self-consistent with the quasiparticle picture of the fluctuation-induced ordering transition. In the approximation of Gaussian (noninteracting) fluctuations, the fluctuative free energy is given by

$$F_{\text{fluct}} = \sum_i \sum_{\mathbf{k}}^{|\mathbf{k}|<\Lambda} \omega^{(i)}_{\mathbf{k}} f^{(i)}_{\mathbf{k}} - T\, \mathrm{S}_{\text{fluct}}, \tag{36}$$

where $\Lambda \approx 2\pi/a$ is the natural cutoff, $f^{(i)}_{\mathbf{k}}$ is equilibrium distribution function, and $\mathrm{S}_{\text{fluct}}$ is the entropy of the noninteracting gas of fluctuations,

$$\mathrm{S}_{\text{fluct}} = \sum_i \sum_{\mathbf{k}}^{|\mathbf{k}|<\Lambda} \left\{ (1 + f^{(i)}_{\mathbf{k}}) \ln(1 + f^{(i)}_{\mathbf{k}}) - f^{(i)}_{\mathbf{k}} \ln f^{(i)}_{\mathbf{k}} \right\} . \tag{37}$$

Note that this approach can be extended to the case of interacting fluctuations. As a general rule, the excitation energy is $\omega_{\mathbf{k}} \ll T_C$, thus the free energy (36) allows further simplification; for low-energy collective excitations (Bose-Einstein statistics), one has

---

[2] The perturbed conical vector for this field configuration is given by $S^x_{\mathbf{k}} = \frac{i}{\sqrt{2}} - \frac{i\ae}{2\sqrt{2}} - \ae - \frac{9i\ae^2}{8\sqrt{2}}$, $S^y_{\mathbf{k}} = \frac{1}{\sqrt{2}} = -i\ae + \frac{\ae}{2\sqrt{2}} - \frac{9\ae^2}{8\sqrt{2}}$ and $S^z_{\mathbf{k}} = 0$. For such a perturbation, both $U\langle S^4 \rangle$ and $A\langle S_x^4 + S_y^4 + S_z^4 \rangle$ are not perturbed even in $\ae^2$. The magnetoelectric response is only $\langle \mathcal{H} \rangle_{\ae}/2Dk_0 = (\mu^2 - 2m^2)\ae - 9\mu^2\ae^2$, which is smaller than for the SkL phase in the phase space under interest.

[3] Here J and D are in proper-energetical units, i.e. with restored M and a.

$$\ln(1+f_{\mathbf{k}}^{(i)}) \simeq \beta\omega_{\mathbf{k}}^{(i)} - \ln\beta\omega_{\mathbf{k}}^{(i)}, \qquad \ln f_{\mathbf{k}}^{(i)} \simeq -\ln\beta\omega_{\mathbf{k}}^{(i)}, \tag{38}$$

where we take into account that the cut-off energy is smaller than the ordering temperature. Thus asymptotically one has

$$F_{\text{fluct}} \simeq T \sum_{\mathbf{k}}^{|\mathbf{k}|<\Lambda} \ln\beta\omega_{\mathbf{k}}. \tag{39}$$

At short length-scales, the collective excitations merge to a single effectively ferromagnetic mode $\omega_{\mathbf{k}} \sim Jk^2$, which gives the main contribution to the free energy. To include the effect of mode-mode interactions, which below T_C promote the propagation of collective excitations in (32) only along the symmetry-broken directions of the underlying phase, we keep the main non-vanishing terms as

$$F_{\text{fluct}}^{\text{short}} \simeq T\sum_{\mathbf{k}} \ln\beta Jk^2(1+\Gamma/Jk^2) \tag{40}$$

$$\simeq T\sum_{\mathbf{k}}(\ln\beta Jk^2 + \Gamma/Jk^2). \tag{41}$$

where

$$\Gamma = \Gamma_1 + \Gamma_2, \quad \Gamma_1 = 4U \sum_{\mathbf{k}',\mathbf{k}''} \delta_{\alpha\beta}\, S^{\alpha}_{-\mathbf{k}''}\, S^{\beta}_{\mathbf{k}-\mathbf{k}'+\mathbf{k}''}, \tag{42}$$

$$\Gamma_2 = 8U \sum_{\mathbf{k}',\mathbf{k}''} S^{\alpha}_{-\mathbf{k}''}\, S^{\beta}_{\mathbf{k}-\mathbf{k}'+\mathbf{k}''}. \tag{43}$$

The first term in (40), $\ln\beta Jk^2$, does not contribute to free energy difference if the helical and skyrmion phases are treated on the same volume in momentum space. The second term gives the fluctuative contribution dependent on the mean-field values of the magnetization in two phases,[4]

$$\Delta F_{\text{fluct}} \approx \Delta F_{\text{fluct}}^{\text{short}} \simeq \frac{5\Lambda UT}{2\pi^2 Jk_0}\left(\langle M^2_{\text{sk}}\rangle - \langle M^2_{\text{con}}\rangle\right) \tag{44}$$

$$= \frac{10}{\pi}\frac{UT}{Da}\left(\langle M^2_{\text{sk}}\rangle - \langle M^2_{\text{con}}\rangle\right). \tag{45}$$

The phase diagram is plotted with the fluctuative energy (44) on top of the mean-field solution, and the described formalism is expected to capture the physics of the system.

D. Justification for the first-order perturbation theory

We finally make a remark that the important physics is already captured in the first order perturbation theory because the dimensionless E field æ is small. The reason why such small E fields can control the skyrmion phase stability so dramatically is that the skyrmion phase competes with the conical phase very close already in the mean-field. By adding the fluctuative contributions, the SkL free energy becomes lower than the conical phase (close to T_C). Such a nontrivial mechanism as we have elucidated here is very useful for applications, since the involved E fields are typical for those used in modern microelectronics.

Nevertheless, for completeness, the second-order perturbation theory is determined by the formula [10]

$$\begin{aligned}|\mathbf{S}_{\mathbf{k}}^{(æ)}\rangle = |\mathbf{S}_{\mathbf{k}}^{(0)}\rangle &+ \sum_{n\neq 0} |\mathbf{S}_{\mathbf{k}}^{(n)}\rangle \frac{\langle \mathbf{S}_{\mathbf{k}}^{(n)}|\hat{\mathcal{H}}_{æ}|\mathbf{S}_{\mathbf{k}}^{(0)}\rangle}{\varepsilon_{\mathbf{k}}^{(0)} - \varepsilon_{\mathbf{k}}^{(n)}} \\ &+ \sum_{n\neq 0}\sum_{m\neq 0} |\mathbf{S}_{\mathbf{k}}^{(n)}\rangle \frac{\langle \mathbf{S}_{\mathbf{k}}^{(n)}|\hat{\mathcal{H}}_{æ}|\mathbf{S}_{\mathbf{k}}^{(m)}\rangle\langle \mathbf{S}_{\mathbf{k}}^{(m)}|\hat{\mathcal{H}}_{æ}|\mathbf{S}_{\mathbf{k}}^{(0)}\rangle}{(\varepsilon_{\mathbf{k}}^{(0)} - \varepsilon_{\mathbf{k}}^{(n)})(\varepsilon_{\mathbf{k}}^{(0)} - \varepsilon_{\mathbf{k}}^{(m)})} \\ &- \sum_{n\neq 0} |\mathbf{S}_{\mathbf{k}}^{(n)}\rangle \frac{\langle \mathbf{S}_{\mathbf{k}}^{(n)}|\hat{\mathcal{H}}_{æ}|\mathbf{S}_{\mathbf{k}}^{(0)}\rangle\langle \mathbf{S}_{\mathbf{k}}^{(0)}|\hat{\mathcal{H}}_{æ}|\mathbf{S}_{\mathbf{k}}^{(0)}\rangle}{(\varepsilon_{\mathbf{k}}^{(0)} - \varepsilon_{\mathbf{k}}^{(n)})^2} \\ &- \sum_{n\neq 0} |\mathbf{S}_{\mathbf{k}}^{(0)}\rangle \frac{\langle \mathbf{S}_{\mathbf{k}}^{(0)}|\hat{\mathcal{H}}_{æ}|\mathbf{S}_{\mathbf{k}}^{(n)}\rangle\langle \mathbf{S}_{\mathbf{k}}^{(n)}|\hat{\mathcal{H}}_{æ}|\mathbf{S}_{\mathbf{k}}^{(0)}\rangle}{2(\varepsilon_{\mathbf{k}}^{(0)} - \varepsilon_{\mathbf{k}}^{(n)})^2}.\end{aligned} \tag{46}$$

It can be verified numerically that the second-order perturbation theory quickly converges. As shown in Fig. 1, for the size of E fields applied experimentally, it is sufficient to use the first-order perturbation theory.

[4] Here we used the discrete summation approximation $\sum_{\mathbf{k}}^{|\mathbf{k}|<\Lambda} \frac{1}{k^2} \approx \int_0^{\Lambda/k_0} \frac{d^3\mathbf{q}}{(2\pi)^3}\frac{1}{q^2} = \frac{\Lambda}{2\pi^2 k_0}$.

[1] J.S. White *et al.*, Phys. Rev. Lett. **113**, 107203 (2014).

[2] M. Janoschek *et al.*, Phys. Rev. B **87**, 134407 (2013)

[3] S. Mühlbauer, B. Binz, F. Jonietz, C. Pfleiderer, A.Rosch, A.Neubauer, R.Georgii, and P.Böni, Science **323**, 915 (2009).

[4] H.S. Green. Matrix mechanics. P. Noordhoff (1965).

[5] T. Arima, J. Phys. Soc. Jpn. **76**, 073702 (2007).

[6] S. Seki, S. Ishiwata, and Y. Tokura. Phys. Rev. B, **86**,

060403 (2012).
[7] Y.H. Liu, Y.Q. Li, and J.H. Han, Phys. Rev. B, **87**, 100402 (2013).
[8] C. Jia, S. Onoda, N. Nagaosa, and J.H. Han, Phys. Rev. B, **76**, 144424 (2007).
[9] M. Belesi, I. Rousochatzakis, M. Abid, U.K. Roler, H. Berger, and J.P. Ansermet, Phys. Rev. B, **85**, 224413 (2012).
[10] A.J. Kruchkov and H.M. Rønnow, arXiv preprint 1702.08863.

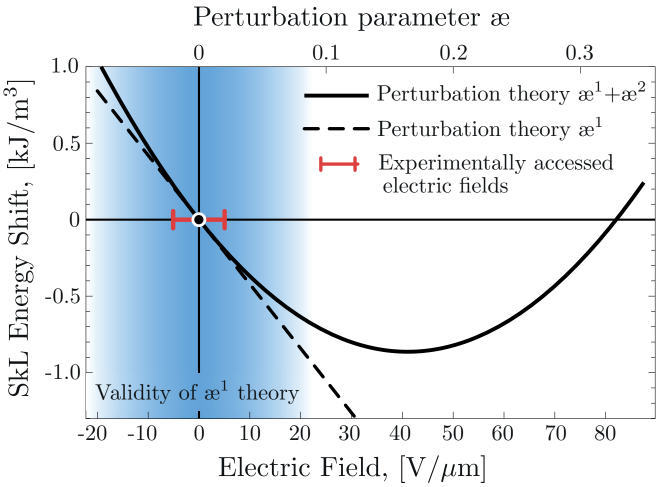

FIG. 1. Shift in the SkL (free) energy in E fields calculated for $E||[111$, $H||[1\bar{1}0]$. Solid line: first + second order (i.e. up to $æ^2$) perturbation theory, dashed line: contribution of the first order ($æ^1$) perturbation theory only. We sketch validity of the $æ^1$ perturbation theory as around $|æ| \sim 0.1$, which for Cu_2OSeO_3 converts to approximately ± 20 V/μm. Experimentally accessed range of electric fields is of order ± 5 V/μm, which is sufficiently captured with the $æ^1$ theory. Figure from Ref.[10].
